# Losartan Prevents Heart Fibrosis Induced by Long-Term Intensive Exercise in an Animal Model

**DOI:** 10.1371/journal.pone.0055427

**Published:** 2013-02-01

**Authors:** Gemma Gay-Jordi, Eduard Guash, Begoña Benito, Josep Brugada, Stanley Nattel, Lluís Mont, Anna Serrano-Mollar

**Affiliations:** 1 Department of Experimental Pathology, Institut d'Investigacions Biomèdiques de Barcelona (IIBB-CSIC), Barcelona, Catalonia, Spain; 2 Institut d'Investigacions Biomèdiques August Pi i Sunyer (IDIBAPS), Barcelona, Catalonia, Spain; 3 Cardiology Department, The Thorax Institute, Hospital Clínic, University of Barcelona, Barcelona, Catalonia, Spain; 4 Research Center, Montreal Heart Institute and Université de Montréal, Montréal, Quebec, Canada; Cardiovascular Research Institute Maastricht, Maastricht University, The Netherlands

## Abstract

**Rationale:**

Recently it has been shown that long-term intensive exercise practice is able to induce myocardial fibrosis in an animal model. Angiotensin II is a profibrotic hormone that could be involved in the cardiac remodeling resulting from endurance exercise.

**Objective:**

This study examined the antifibrotic effect of losartan, an angiotensin II type 1 receptor antagonist, in an animal model of heart fibrosis induced by long-term intense exercise.

**Methods and Results:**

Male Wistar rats were randomly distributed into 4 experimental groups: Exercise, Exercise plus losartan, Sedentary and Sedentary plus losartan. Exercise groups were conditioned to run vigorously for 16 weeks. Losartan was orally administered daily before each training session (50 mg/kg/day). Time-matched sedentary rats served as controls. After euthanasia, heart hypertrophy was evaluated by histological studies; ventricular collagen deposition was quantified by histological and biochemical studies; and messenger RNA and protein expression of transforming growth factor-β1, fibronectin-1, matrix metalloproteinase-2, tissue inhibitor of metalloproteinase-1, procollagen-I and procollagen-III was evaluated in all 4 cardiac chambers. Daily intensive exercise caused hypertrophy in the left ventricular heart wall and originated collagen deposition in the right ventricle. Additionally long-term intensive exercise induced a significant increase in messenger RNA expression and protein synthesis of the major fibrotic markers in both atria and in the right ventricle. Losartan treatment was able to reduce all increases in messenger RNA expression and protein levels caused by exercise, although it could not completely reverse the heart hypertrophy.

**Conclusions:**

Losartan treatment prevents the heart fibrosis induced by endurance exercise in training animals.

## Introduction

In recent years, a growing concern has arisen from numerous observational studies reporting that long-term intense sport activity could be associated with an increased risk of cardiac arrhythmias [1]–[5]. In a previous study by our group we have demonstrated a profibrotic cardiac remodeling following long-term intensive exercise training in experimental animals, this could be the basis for an increased arrythmogenicity in athletes [6]. In this respect, a well designed recently published study, relates the presence of myocardial fibrosis in veteran endurance athletes in the absence of any other cause [7]. The etiology and clinical significance of these findings have yet to be fully elucidated. Theres is therefore a need for better understanding of the biological process involved and of any upper limit above which intense exercise can be harmful to health as well as the possibility of taking action to avoid this damage.

Long-term exercise is associated with hemodynamic changes and alters the loading conditions of the heart, these changes have classically characterized the physiology of “athlete's heart”. It is known that hemodynamic overload activates the renin-angiotensin system (RAS) in the heart [8], [9]. Although RAS plays an important role in cardiovascular homeostasis by influencing vascular tone, fluid and electrolyte balance, experimental evidence suggests that RAS activation induces fibroblast proliferation and myocyte hypertrophy [10], [11]. Angiotensin II (ANGII) is produced by proteolytic cleavage of its precursor angiotensin I by angiotensin converting enzyme (ACE). ANGII plays an important role in cardiac fibrogenesis [12] by acting as a potent grown factor and cytokine for vascular smooth muscle cells, cardiac myocytes and cardiac fibroblasts, via activation of the angiotensin type I receptor (AT1) [13], [14].

Losartan is a selective AT1 receptor antagonist. It has been demonstrated that inhibition of cardiac RAS with ACE inhibitors and/or ANGII receptor blockers improves left ventricle function, prevents geometric remodeling, and prolongs survival in several heart diseases, such as hypertension, heart failure, ischemic heart disease, and diabetes mellitus [15], [16]. The antifibrotic effect of losartan has also been described in other fibrotic processes such as pulmonary fibrosis [17].

The main objective of this study was to evaluate the anti-fibrotic effect of losartan in an animal model of heart fibrosis induced by chronic endurance exercise. Our results showed that losartan was able to prevent the development of myocardial fibrosis caused by intense exercise.

## Materials and Methods

### The experimental design

This study conformed to European Community (Directive 86/609/EEC) and Spanish guidelines for the use of experimental animals and it was approved by the institutional committees of animal care and research. 24 pathogen-free, 4 week old, male Wistar rats, weighing 100–125 g at the beginning of experiments (Charles River Laboratories, France), were housed in a controlled environment (12 ∶ 12-h light-dark cycle), and fed rodent chow (A04; Panlab, Barcelona, Spain) and tap water *ad libitum*.

#### Experimental groups

Animals were randomly distributed into four experimental groups:
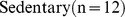


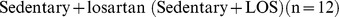


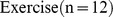


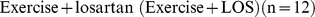
The exercise protocol was performed as in our previous study [6]. Briefly, exercise rats underwent a daily running session on a treadmill (Panlab, Barcelona, Spain) five days a week for 16 weeks. Losartan (50 mg/kg/day) was administered orally, every day at the same hour, starting in the first exercise session and continuing up to the end of the experiment. The drug was dissolved in a final volume of 1,5 ml of distilled water. Oral administration was selected as this is the usual method in a clinical setting. The first exercise session lasted for 10 min at 25 cm/s, and the duration and intensity were gradually increased reaching 60 min at 60 cm/s on the 10^th^ day these levels were then maintained until the end of training. The animals were euthanized three days after the end of the training program to avoid any immediate responses. The hearts were quickly removed, weighed, dissected into left ventricle (LV), right ventricle (RV), left atrium (LA) and right atrium (RA); they were then frozen in liquid nitrogen for storage at −80°C until analyzed (n = 6, for each group), or fixed for histological studies (n = 6, for each group).

### Cardiac hypertrophy

**Figure 1:**
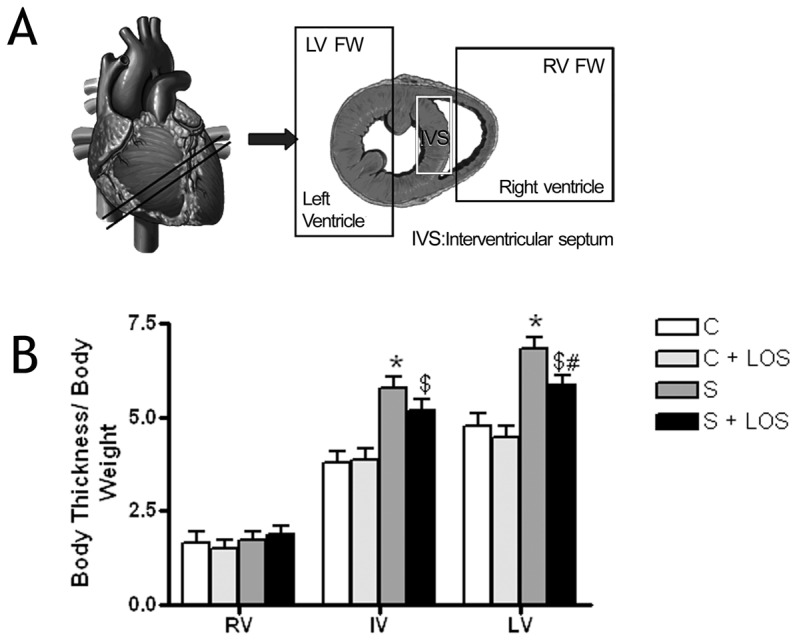
Assessment of ventricular hypertrophy. (A) Representative graph of a tissue slice indicating the three areas of study: right ventricular free wall (RV FW), left ventricular free wall (LV FW) and interventricular septum (IVS). Twelve measures were obtained from each of the areas. (B) Results of ventricular wall thickness indexed for body weight in all the experimental groups. LV FW was significantly higher in all exercise groups compared to sedentary groups. Data are means ± SEM of 6 (sedentary and sedentary+LOS) and 6 (exercise and exercise+LOS). * p<0.05 sedentary *vs* exercise; $ p<0.05 sedentary+LOS *vs* exercise+LOS, and, # p<0.05 exercise *vs* exercise+LOS.

Cardiac hypertrophy was assessed by determining the heart-weight to body-weight ratio and by measuring the ventricular wall thickness at papillary muscle levels. To measure the wall thickness from RV free wall (RV FW), interventricular septum (IV), and LV free wall (LV FW), 12 measurements from each wall were taken per animal in heart paraffin sections using analySIS Image Processing software (Soft Imaging System GMBH, Germany) (Fig. 1A). Differences in ventricular size were controlled by indexing wall-thickness to body-weight.

### Histology and morphometry

For the histological studies, the heart was perfused with a fixative solution (10% neutral-buffered formalin) at a pressure of 80 cm H_2_O, immersed in the fixative for 12–24 h, and embedded in paraffin. Sections were cut at 4 µm thick serial sections and stained with Picrosirius red to identify connective tissue and collagen deposition. Additionally, sections from the RV were stained with picrosirius-red to quantify of collagen deposition using analySIS Image Processing software (Soft Imaging System GMBH, Germany) as previously described [6]. Perivascular collagen was excluded from this measurement.

### mRNA analysis

Total RNA was extracted from 50 to 100 mg of a section of the LV, RV, LA and RA using Trizol® reagent (Invitrogen Corporation, CA, USA) according to the manufacturer's protocol. RNA integrity and loading amounts were assessed by examining UV/VIS at multiple wave lengths following the ND-3300 user manual V2.5, instructions (ND-3300, NanoDrop Technologies, USA). Analysis of transforming growth factor-β1 (TGF-β1), fibronectin-1, metalloproteinase 2 (MMP-2), tissue inhibitor of metalloproteinase 1 (TIMP-1), procollagen-1 (Proc-I) and procollagen-3 (Proc-III) mRNA expression was obtained by Real-Time PCR. One µg total mRNA was converted to cDNA with the iScript cDNA (Bio-Rad Laboratories, CA, USA), according to the manufacturer's protocol. 100 ng of cDNA was amplified by the iCycler IQ™ version 3.1 (Bio-Rad Laboratories, CA, USA) using Applied Biosystems (Applied Biosystems, CA, USA) TaqMan gene expression assays (Rn00572010-m1 for TGF-β1, Rn00569575-m1 for fibronectin-1, Rn02532334-s1 for MMP-2, Rn00587558-m1 for TIMP-1, Rn01526721-m1 for procollagen-1, Rn01437675-m1 for procollagen-3 and Rn00667869-m1 for actin, which was used as a housekeeping reference). Data was analyzed with the ΔCt method.

### SDS-PAGE and Western blot

Protein samples were extracted using Nonidet P-40 buffer. SDS-PAGE was performed on 5%–13% acrylamide gels. Proteins were electrotransferred to nitrocellulose membrane and probed with primary antibodies. The antibodies used included mouse monoclonal anti-TGF-β1 (ab27969, dilution1/2000), mouse monoclonal anti-MMP2 (ab7032 dilution 1/1000), rabbit polyclonal to TIMP1 (ab61224 dilution 1/1000), mouse monoclonal to collagen-I (ab6308 dilution 1/1000) (all of them acquired from Abcam plc, Cambridge, UK); rabbit polyclonal antifibronectin (BP8025, dilution 1/1000) (Acris Antibodies GmbH, Herford, Germany) and rabbit polyclonal to collagen-III (dilution 1/500) (Santa Cruz Biotechnology, Ca, USA), and mouse monoclonal anti-actin (dilution (1/1000) (Chemicon-Millipore Co, MA, USA), which served as a housekeeping reference. The membranes were incubated with the corresponding peroxidase-conjugated secondary antibodies, washed, and then incubated with ECL reagents (GE Healthcare Europe GmbH; Freigburg; GE) before exposure to high-performance chemiluminescence films. Gels were calibrated using Bio-Rad standard proteins (Hercules, CA) with markers covering a 7–240 kDa range. Films were scanned by using image-editing software NIH ImageJ software for densitometric analysis of immunoreactive bands.

### Statistical analysis

Data is expressed as mean ± SEM values with 95% confidence intervals. Statistical analysis was carried out by analysis of variance (ANOVA) followed by appropriate post-hoc tests, including Bonferroni correction and unpaired t-test. (GraphPad Software Inc, San Diego, CA, USA). Significance was accepted when p<0.05.

## Results

### Cardiac hypertrophy

Exercise causes a significant body weight loss in both exercise groups. Losartan treatment did not modify the body weight loss. Heart weight did not show any significant differences between all the study groups. However, the heart-weight-to-body-weight ratio was significantly different between exercise groups and sedentary groups. The body and heart weight, and the ratio between body and heart weight are summarized in Table 1.

**Table 1:** Body weights, heart weights and heart weight/body weight ratios at the endpoint.

	Body Weight	Heart Weight	Heart Weight/Body Weight (×1000)
Sedentary	414.8±13.98	1.027±0.090	0.00244±0.00009
Sedentary+LOS	382.5±17.97	0.940±0.090	0.00245±0.00025
Exercise	357.1±8.25*	1.118±0.050	0.00308±0.00006*
Exercise+LOS	339.7±8.96†	1.025±0.042	0.00312±0.00004†

Groups: Sedentary; Exercise, Sedentary+LOS = sedentary plus losartan; Exercise+LOS = exercise plus losartan. BW = body weight; HW = heart weight; HW/BW = heart weight-to-body weight ratio. Data are mean ± SEM of 6 animals (sedentary and sedentary+LOS) and 6 animals (exercise and exercise+LOS).

* p<0.05 exercise group *vs* sedentary group and

† p<0.05 sedentary+LOS *vs* exercise+LOS.

Direct measurements of wall thickness confirmed significant increments in IV and LV FW in the exercised groups compared to their respective sedentary groups, exercise+LOS shows a reduction in the LV FW thickening compared to exercise group, although the exercise+LOS remained significantly increased relative to LOS alone. No significant differences were observed in RV FW thickness (Fig. 1B).

### Histopathology

**Figure 2:**
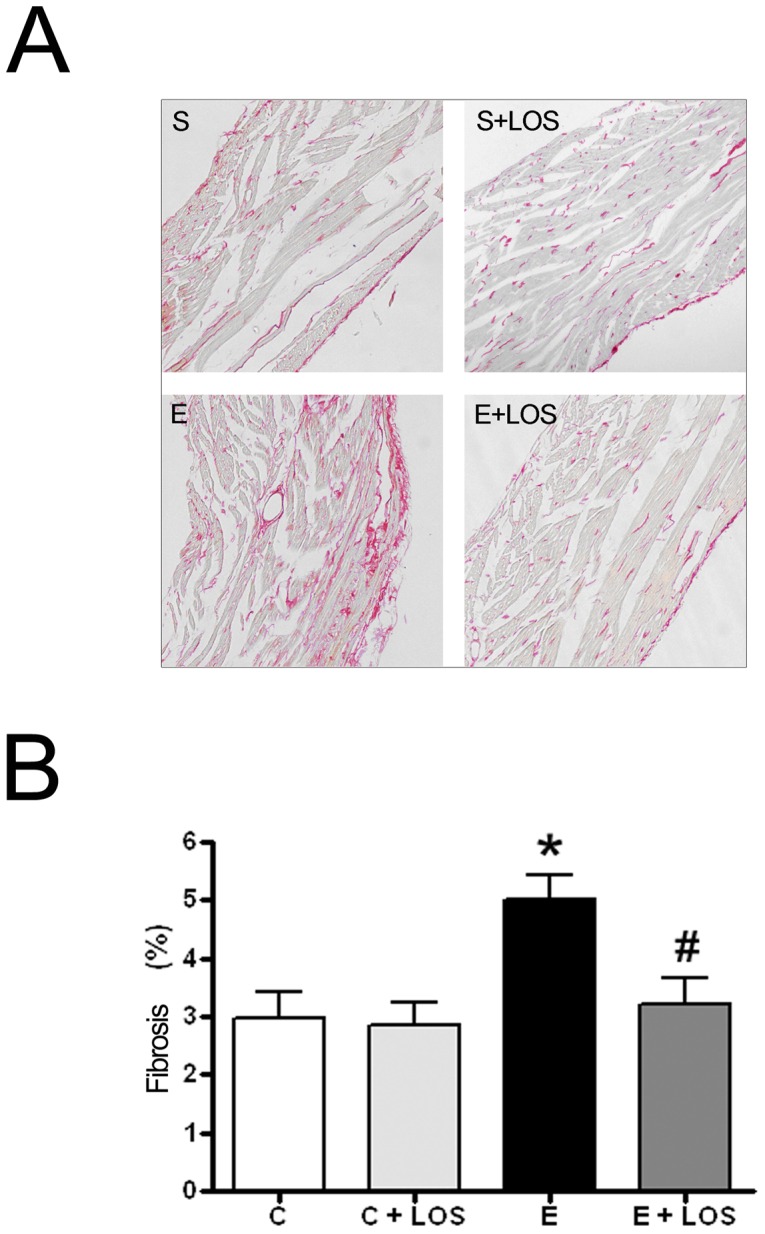
Assessment of right ventricle myocardial fibrosis. (A) Representative Picrosirius red-stained photomicrographs of the right ventricle, S: sedentary; S+LOS: sedentary plus losartan; E: exercise; E+LOS: exercise+losartan. Magnification ×200. An excess of interstitial collagen deposition (red staining) was observed in the right ventricle of the exercise group. (B) Morphometryc analysis of collagen deposition. Mean ± SEM collagen content in RV. * p<0.05 sedentary *vs* exercise, # p<0.05 exercise *vs* exercise+LOS.

To further examine the effect of exercise heart serial sections were stained with Picrosirius red to identify collagen deposition, and examined by light microscopy. Diffuse interstitial collagen deposition associated with disturbances in myocardial architecture was observed only in the exercise group (Fig. 2A). Additionally, morphometric quantification in RV confirmed a significant increase in collagen deposition only in the exercise group. No differences in collagen density were observed in the exercise+LOS group. (Fig. 2 B)

### mRNA analysis

Messenger RNA (mRNA) expression of TGFβ-1, fibronectin-1, MMP-2, TIMP-1, procollagen-1 and prollagen-3 was measured in all cardiac chambers of rats in all the different groups under study.

**Figure 3:**
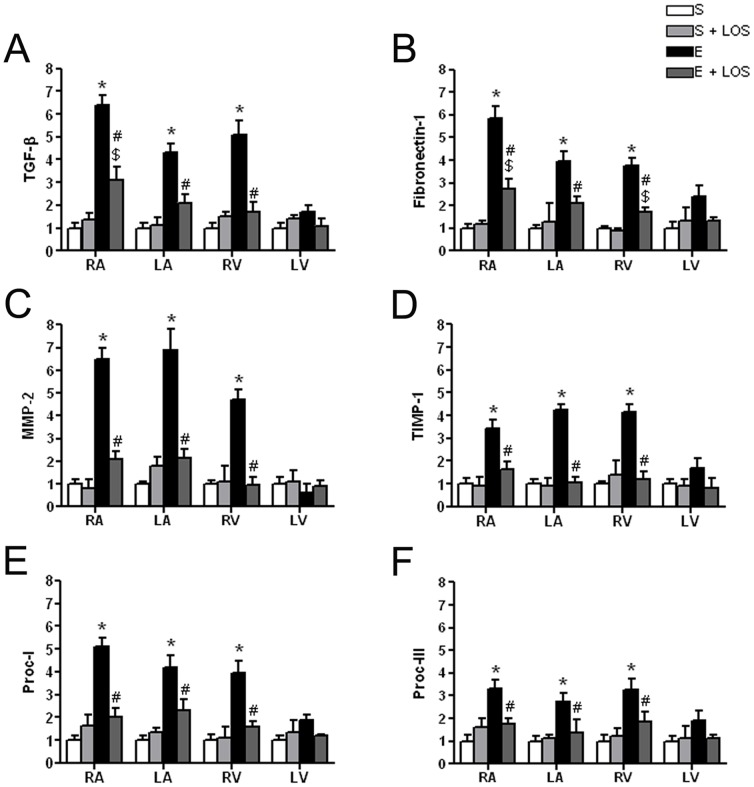
mRNA expression of fibrosis markers in each of the four cardiac chambers. Results are expressed according to the ΔCt method, with exercise group values in reference to those of the sedentary group (ΔCt ± SEM). Data are means ± SEM of 4 animals (all groups). * p<0.05 sedentary *vs* exercise; $ p<0.05 sedentary+LOS vs exercise+LOS, and, # p<0.05 exercise *vs* exercise+LOS.

TGFβ-1 expression was significantly increased in the RA of exercise and exercise+LOS compared to its respective sedentary groups, although the increase observed in the exercise+LOS group were significantly reduced compared to that of the exercise group (Figure 3A). Furthermore, TGF-β1 expression was also significantly increased in the LA and RV in only the exercise group compared to the sedentary group, while exercise+LOS did not show any significant differences compared to its sedentary group (Figure 3A).

Fibronectin-1 expression was significantly increased in RA and RV of exercise and exercise+LOS, compared to its respective sedentary groups, although the increase showed by exercise+LOS was significantly reduced compared to the exercise group (Figure 3B). Fibronectin-1 expression was also increased in the LA in only the exercise group compared to the sedentary group, while exercise+LOS did not show any significant differences compared to its sedentary group.

Finally, MMP-2, TIMP-1, procollagen-I and procollagen-III mRNA expression was significantly increased in the RA, LA and RV only in the exercise group, while its expression in the exercise+LOS group was similar to that of the control groups (Figure 3C, 3D, 3E and 3F).

### Western blot analysis

Alterations in protein expression corresponding to mRNA changes were assessed by Western blot analysis for TGF-β1, fibronectin-1, MMP-2, TIMP1, collagen-I, and collagen-III.

**Figure 4:**
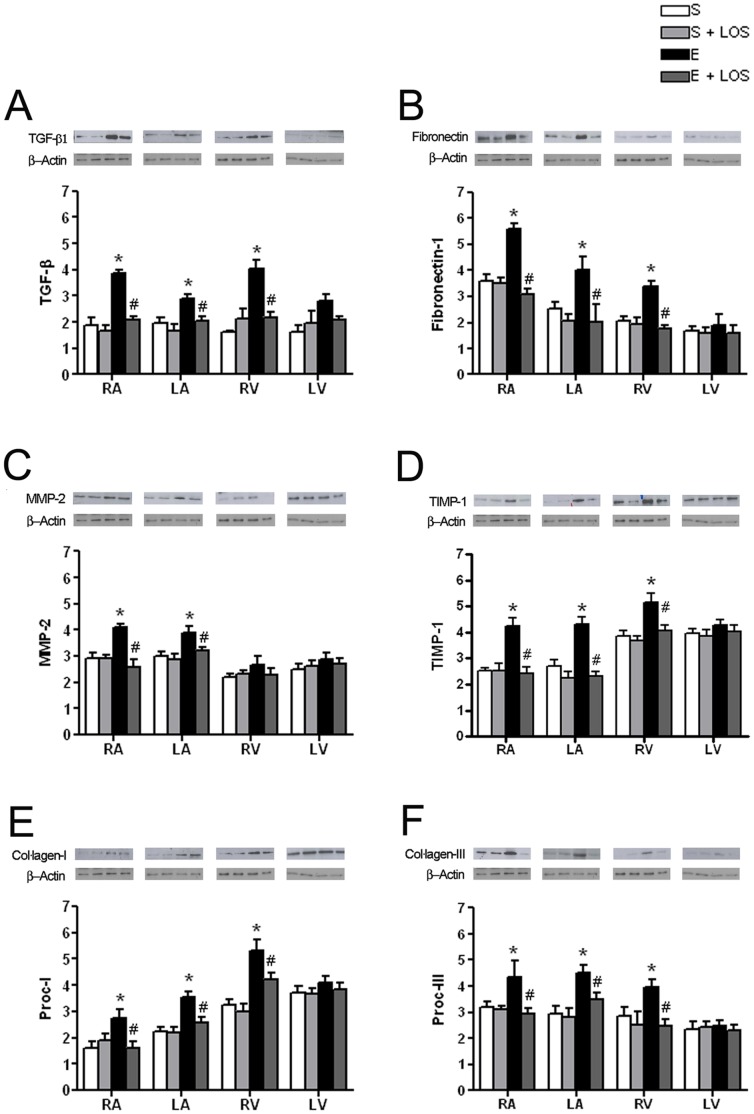
Protein levels of fibrotic markers in each one of the four cardiac chambers. Mean ± SEM protein levels of fibrotic markers (A) TGF-β1, (B) Fibronectin-1, (C) MMP-2, (D) TIMP-1, (E) collagen-1, (F) collagen-III analyzed by immunoblot (examples shown above bar graphs) and normalized to β-Actin. (All groups n = 4).

TGF-β1 protein levels were significantly increased in both atria and in the RV of exercised rats. Exercise+LOS group showed similar levels to the sedentary groups (Figure 4A).

Fibronectin-1 protein levels showed significant increases in RA, LA and RV in the exercise group, while the exercise+LOS group showed the same patterns of protein levels as the sedentary groups (Figure 4B).

MMP-2 protein synthesis was significantly increased in the RA and in the LA of the exercise group, whereas the synthesis levels for MMP-2 did not show any differences between the exercise+LOS and the sedentary groups (Figure 4C).

TIMP1 showed increased synthesis in the RA, LA and RV of the exercise group, losartan treatment was able to avoid such increases in the exercise+LOS group (Figure 4D).

Collagen-I protein levels were significantly greater in the RA, LA and RV of the exercise group; exercise+LOS showed protein levels similar to those of sedentary group (Figure 4E).

Finally, collagen-III protein levels showed an increase in the exercise group in RA, LA and RV, while the exercise+LOS group showed the same protein levels as the sedentary groups (Figure 4F). Overall these results confirm the development of significant changes in the extracellular matrix (ECM) changes after 16 weeks of intensive endurance exercise, with a clear fibrosis development in the RV but not the LV, while also corfirming the effectiveness of losartan treatment in preventing this pathological remodeling.

## Discussion

We have shown that losartan, a selective AT1 receptor antagonist, ameliorates experimental exercise-induced myocardial fibrosis. Our findings provide further evidence for the anti-fibrotic effect of losartan.

Chronic exercise induces hemodynamic changes and alters the loading conditions of the heart [6], thus generating structural cardiac adaptations that are considered physiological, (and commonly known as athlete's heart) [18]. Long-term volume and pressure overloads, caused by intense exercise, can induce cardiac maladaptative responses including remodeling and fibrosis, which ultimately lead to heart tissue dysfunction similar to that found in pathological conditions of chronic overload such as heart failure and hypertension [19], [20]. Several studies with animal models of acute exercise have shown histological evidence of reactive scar tissue formation and elevation of necrosis and fibrosis markers immediately following intense exercise [21], [22]. Furthermore, Lindsay et al have found biochemical evidence of myocardial fibrosis in veteran endurance athletes [23]. Also, in a previous animal model we have demonstrated the direct relationship between a long-term endurance exercise schedule and the development of myocardial fibrosis and increased arrhythmia inducibility [6]. In this study, the progressive alteration in ECM composition induced by exercise increased myocardial stiffness, promoted the development of diastolic dysfunction and became a potential substrate for the development of arrhythmias [6]. It is particularly noteworthy that our findings in an animal model were similar to those observed in a study on veteran endurance athletes [7].

The present results confirm that 16 weeks of endurance exercise training in rats promotes left ventricular hypertrophy [6]. Furthermore, exercised animals developed myocardial fibrosis and showed alterations in ECM protein synthesis in both atria and in the right ventricle, indicating that these changes could promote alterations in heart functionality [6]. Therefore, ameliorating and preventing myocardial fibrosis would be very important in the management of future cardiovascular events [24], [25]. ANGII is a powerful stimulus for progressive cardiac remodeling and plays a significant role in promoting myocardial fibrosis [26], [27]. AT1 receptor antagonists have antifibrotic and anti-growth effects on the myocardium in experimental settings [28], [29]. It has been reported that circulating ANG II plays a central role in the progression of myocardial fibrosis and hypertrophy in hypertensive patients [30], [31].

As described in with previous works [6], [32], our results also showed an increased cardiac mass and left ventricular hypertrophy, reflecting the enlargement of the left ventricular cavity that following long-term endurance training and that is considered typical of the athlete's heart [18]. Left ventricular hypertrophy as detected by echocardiogram is an independent predictor of morbidity and mortality in subjects with high blood pressure [33] and in the population at large [34]. In the literature, there are several studies evaluating the therapeutic benefits of ACE inhibitors or the intervention of AT1 antagonists in reducing left ventricular hypertrophy [35], [36]. In agreement with these studies, our findings show that losartan treatment was able to partially prevent left ventricular hypertrophy in exercised animals. One interesting observation is the fact that the development of hypertrophy is observed in the LV, but the increases in both the expression and the synthesis of fibrotic markers are observed in the RA, LA and RV. This could be explained by the intrinsic features of these cavities, including their thinner walls, which could make them more susceptible to damage from heart volume and pressure overload induced by exercise. In contrast, the LV structure is thicker and responds to pressure and volume overload by increasing its thickness even further, thus demonstrating greater resistance to changes in the composition of the ECM. Cardiac remodeling in response to hemodynamic stress and/or injury includes changes in the cardiomyocyte compartment as well as the nonmyocyte compartment (endothelial cells, vascular smooth muscle cells, macrophages, and cardiac fibroblasts). Pathological changes in cardiac remodeling include myocyte hypertrophy, myocyte death, and dynamic changes in the interstitium. Interstitial changes involve qualitative and quantitative alterations in the fibrillar collagen network (composed mainly of types I and III fibrillar collagen), including changes in collagen cross-linking [37]. Collagen-I determines the stiffness of cardiac muscle, whereas collagen-III is more distensible. Thus, the ratio of collagen-I to collagen-III can be a marker of the ECM determinants of cardiac stiffness [38]. We observed a significant increase in collagen-I and III protein expression in the RV in the exercise group, where collagen-I expression was more prominent, indicating that long-term, intensive exercise could increase cardiac stiffness. Losartan treatment was able to avoirevent all the increases in fibrotic markers caused by endurance exercise, thereby preventing myocardial ECM remodeling. The antifibrotic effect of AT1 receptor blockade has been reported in other studies [29], [39]. Our findings agree with those of Chen et al [37], who showed that an AT1 receptor antagonist prevented the early induction of TGF-β1 and the subsequent development of cardiac fibrosis, indicating that ANG II acts indirectly through the expression of TGF-β1 to induce cardiac fibrosis. It has been proposed that a phenotypic change from the cardiac fibroblast to the more synthetic myofibroblast phenotype takes place under the influence of cytokines such as TGF-β1, promoting the deposition of collagen [40]. Myocardial collagen content is tightly regulated by a balance between collagen production and degradation. Extracellular degradation of collagen is the most important brake on collagen metabolism and it is effected by matrix metalloproteinases (MMPs) [41]. The intramyocardial coronary arterioles also undergo changes in cardiac remodeling resulting in medial thickening and perivascular fibrosis. Collectively these changes lead to progressive impairment of the cardiac function. Our results showed a significant increase in mRNA expression for TGF-β1, MMP-2, TIMP-1, fibronectin-1, collagen type I and collagen type III as a consequence of intense and long-lasting exercise. Losartan treatment was able to bring all these increases down to control levels in this animal model, indicating the beneficial effect of blocking the RAS system in preventing on myocardial remodeling resulting from intense exercise.

In summary, this study demonstrates that losartan treatment in exercised animals prevents the heart fibrosis induced by endurance exercise and strengthens the recent theory that intense exercise could be associated with left ventricular hypertrophy and interstitial myocardial fibrosis [6], [7], [23], [42]. This finding opens up the possibility of using losartan as a treatment for preventing of the heart's maladaptative responses to long-term intense exercise.
